# Development and Validation of Prognostic Nomogram for Postpartum Hemorrhage After Vaginal Delivery: A Retrospective Cohort Study in China

**DOI:** 10.3389/fmed.2022.804769

**Published:** 2022-03-07

**Authors:** Zixuan Song, Xiaoxue Wang, Yangzi Zhou, Yuting Wang, Dandan Zhang

**Affiliations:** ^1^Department of Obstetrics and Gynecology, Shengjing Hospital of China Medical University, Shenyang, China; ^2^Department of Health Management, Shengjing Hospital of China Medical University, Shenyang, China

**Keywords:** nomogram, postpartum hemorrhage, vaginal delivery, prediction model, logistic regression analyses logit

## Abstract

**Background:**

Postpartum hemorrhage (PPH) is a common complication following vaginal delivery and in severe cases can lead to maternal death. A straightforward predictive model is required to enable prenatal evaluations by obstetricians to prevent PPH complications.

**Methods:**

Data of patients who delivered vaginally after 37 weeks of gestation were retrospectively collected from the medical database at Shengjing Hospital of China Medical University for the period 2016 to 2020. PPH was defined as blood loss of 500 mL or more within 24 h of delivery, and important independent prognostic factors were determined using univariate and multivariate logistic regression analyses to construct nomograms regarding PPH.

**Results:**

A total of 24,833 patients who delivered vaginally were included in this study. The training cohort included 22,302 patients who delivered between 2016 and 2019 and the external validation cohort included 2,531 patients who delivered during 2020. Nomogram was created using data such as age, race, occupation, parity, gestational weeks, labor time, neonatal weight, analgesic delivery, gestational diabetes mellitus, premature rupture of membranes, anemia, hypertension, adenomyosis, and placental adhesion. The nomogram has good predictive power and clinical practicality through the analysis of the area under the curve and decision curve analysis. Internal verification was performed on the nomogram for PPH, demonstrating consistency between the nomogram's predicted probability and actual probability.

**Conclusions:**

The developed and validatable nomogram is a good predictor of PPH in vaginal delivery and can be used in clinical practice to guide obstetricians to administer preventive therapies before delivery.

## Introduction

Severe postpartum hemorrhage is the main cause of maternal morbidity and mortality worldwide. According to the data released by the World Health Organization (WHO) (1), 25% of the total annual maternal deaths worldwide are caused by postpartum hemorrhage (PPH). Furthermore, over 80% of PPH cases occur within 2 h following delivery. PPH is characterized by rapid occurrence and development, and can lead to haemorrhagic shock, diffuse intravascular coagulation, infection, postpartum anemia, and other complications in a short time. Severe cases require hysterectomy or can result in maternal death (2). In addition, complications such as puerperal infection, post-transfusion diseases, postpartum psychological diseases, and Sheehan's syndrome could still occur in surviving parturients after successful rescue, which can seriously impact their quality of life.

It is established that PPH is associated with four main causes: uterine atony, placental complications, laceration of the soft birth canal, and coagulation dysfunction. During normal delivery, placental dissection and delivery is promoted through uterine contractions which close the blood sinuses on the placental dissection by squeezing the muscle fibers of the uterine muscle layer, thereby further stimulating the coagulation function. By accumulating large numbers of platelets to plug the surface of the placental dissection, a cascade of mechanisms develops to avoid PPH (3). During this process, any factor that is affected could cause severe PPH and these factors mutually influence and reinforce each other. Recent reports indicate that in addition to the four main independent factors, there are other previously unknown factors that may affect PPH, such as membrane adhesion, residual placenta, prolonged labor, uterine fibroids, and history of cesarean section.

Rossen et al. suggest that severe PPH can be prevented (4). In 2015, the American College of Obstetricians and Gynecologists issued the “Consensus on the Safe management of PPH” (5) during pregnancy and childbirth, which provides comprehensive guidelines regarding PPH. These recommendations have elevated PPH from simple management to management according to the consensus; diversified and individualized clinical management principles should be adopted to guide clinical practice according to the needs of individual patients and the resources of the medical institutions.

Currently, nomograms are widely used in the prognosis of diseases to aid clinicians in decision makings. In recent years, this tool has garnered increased attention of obstetricians and gynecologists (6, 7). To the best of our knowledge, there is currently no nomogram for predicting bleeding after vaginal delivery alone. In this study, we evaluated the factors related to PPH based on the data of patients who delivered vaginally at our center, and constructed a nomogram for PPH.

## Methods

### Patients

Data of patients who delivered vaginally after 37 weeks of gestation were retrospectively retrieved from the medical database at Shengjing Hospital of China Medical University from 2016 to 2020. The patients who delivered in 2020 were included in the external validation cohort, and the remaining patients were included in the training cohort. The research protocols were approved by the Ethics Committee of the Shengjing Hospital of China Medical University (No. 2021PS744K).

### Data Collection

PPH was defined as blood loss of 500 mL or more within 24 h of delivery. The quantity of blood was measured by volume and weighing methods: Immediately after delivery of the fetus, a special curved plate was placed under the maternal hip and removed 2 h following delivery; the volume of blood in the curved plate was measured using a measuring cup. Two hours later, a special perineal pad was placed under the puerperal hip to collect blood until 24 h after delivery. The perineal pad could be replaced many times for patients with considerable blood loss. The total weight of the perineal pad was calculated by the weight difference of the perineal pad before and after blood collection (g), and this value was divided by 1.05 g/ mL to calculate blood loss (mL). Finally, the 24 h postpartum blood loss comprised the total amount of blood collected in the curved plate and perineal pad (s). All patients' clinical data were retrospectively collected from the hospital information system. According to the Guidelines for Data Processing and Analysis of the International Physical Activity Questionnaire (IPAQ), patients' work intensity during pregnancy was divided into three grades: low, moderate, and high (8).

Demographic factors and clinical characteristics were evaluated to screen for risk factors affecting PPH. Demographic factors included age, race, and education. The clinical factors included gestational time, delivery time, gestational age, and labor time. X-tile software version 3.6.1(Yale University School of Medicine, New Haven, CT, USA)(9) was used to evaluate appropriate cut-off values for continuous variables such as gestational weeks and labor time in combination with commonly used critical clinical values, and continuous variables were grouped for statistical analysis (Supplementary Materials S1–S5).

In this study, we used the X-Tile software in combination with the clinically common cut-off value method for stratifying the continuous variables to better distinguish the risk of PPH. For age, the best cut-off for X-tile was 29 years. However, in practice, we consider patients older than 35 years as older pregnant women. Therefore, we set the cut-off values for age at 29 and 35 years. The best cut-off value predicted by the X-Tile software for the weight of newborns was 3,860 g. In clinical practice, we treat newborns weighing more than 4,000 g as macrosomia. Therefore, we set the cut-off values for newborn weight as 3,860 g and 4,000 g. The stratification method combining X-Tile software and clinical cut-off values takes into account both statistical significance and clinical practicability of the data, enabling convenient clinical application of the nomogram.

### Statistical Analysis

All data in the RStudio environment were analyzed using R version 3.6.3 (R Foundation for Statistical Computing, Vienna, Austria, http://www.r-project.org). Based on univariate and multivariate logistic regression analyses of the risk factors that may affect PPH in the training cohort, a nomogram of PPH was constructed to predict the risk of PPH. Nomograms were evaluated by assessing the area under the ROC curve (AUC) (10). The nomograms were internally calibrated using bootstrapping (1,000 resamplings). The nomogram was externally calibrated using validation queues. The clinical efficacy of the nomogram was evaluated by decision curve analysis (DCA) (11), and the net benefit for each risk threshold probability was calculated. Statistical significance (*P*) was set at <0.05.

## Results

### Patient Characteristics

A total of 27,389 patients who delivered vaginally at Shengjing Hospital of China Medical University from 2016 to 2020 were eligible. Of these, 24,833 patients fulfilled the inclusion criteria. The training cohort included 22,302 patients delivered between 2016 and 2019 whereas the external validation cohort involved 2,531 patients delivered in 2020. PPH occurred in 1,468 patients (6.6%) in the training cohort. In the external validation cohort, 155 patients (6.1%) experienced PPH. Specific patient characteristics are shown in Table 1.

**Table 1:** Patient characteristics.

	**Training cohort (*****N*** **=** **22,302)**		**External validation cohort (*****N*** **=** **2,531)**	
	**No postpartum hemorrhage (*N* = 20,834)**	**Postpartum hemorrhage (*N* = 1,468)**	**No postpartum hemorrhage (*N* = 2,376)**	**Postpartum hemorrhage (*N* = 155)**
**Age (years)**				
≤29	11,272 (54%)	697 (47%)	1,026 (43%)	50 (32%)
30–34	8,272 (40%)	686 (47%)	1,161 (49%)	91 (59%)
≥35	1,290 (6.2%)	85 (5.8%)	189 (8.0%)	14 (9.0%)
**Race**				
Han nationality	18,750 (90%)	1,249 (85%)	2,098 (88%)	125 (81%)
Ethnic minorities	2,084 (10%)	219 (15%)	278 (12%)	30 (19%)
**Education**				
Middle school or below	1,818 (8.7%)	95 (6.5%)	242 (10%)	12 (7.7%)
High school	5,422 (26%)	344 (23%)	660 (28%)	42 (27%)
Undergraduate	11,456 (55%)	845 (58%)	1,257 (53%)	81 (52%)
Graduate or above	2,138 (10%)	184 (13%)	217 (9.1%)	20 (13%)
**Occupation**				
Heavy physical	9,416 (45%)	406 (28%)	780 (33%)	33 (21%)
Moderate physical	9,122 (44%)	836 (57%)	1,319 (56%)	96 (62%)
Light physical	2,296 (11%)	226 (15%)	277 (12%)	26 (17%)
**Pregnancy**				
1	12,650 (61%)	970 (66%)	1,264 (53%)	101 (65%)
2	5,434 (26%)	328 (22%)	719 (30%)	32 (21%)
≥3	2,750 (13%)	170 (12%)	393 (17%)	22 (14%)
**Parity**				
0	16,110 (77%)	1,318 (90%)	1,812 (76%)	142 (92%)
≥1	4,724 (23%)	150 (10%)	564 (24%)	13 (8.4%)
**Gestational weeks**				
<40	12,388 (59%)	676 (46%)	1,402 (59%)	64 (41%)
≥40	8,446 (41%)	792 (54%)	974 (41%)	91 (59%)
**Duration of first stage of labor (min)**				
≤303	11,899 (57%)	535 (36%)	1,472 (62%)	62 (40%)
>303	8,935 (43%)	933 (64%)	904 (38%)	93 (60%)
**Duration of second stage of labor (min)**				
≤68	17,896 (86%)	1,014 (69%)	2,009 (85%)	94 (61%)
>68	2,938 (14%)	454 (31%)	367 (15%)	61 (39%)
**Third stage of labor (min)**				
≤9	18,684 (90%)	1,123 (76%)	2,134 (90%)	113 (73%)
>9	2,150 (10%)	345 (24%)	242 (10%)	42 (27%)
**Neonatal weight (g)**				
≤3,860	18,834 (90%)	1,059 (72%)	2,174 (91%)	112 (72%)
3,861–4,000	1,003 (4.8%)	108 (7.4%)	98 (4.1%)	12 (7.7%)
>4,000	997 (4.8%)	301 (21%)	104 (4.4%)	31 (20%)
**Time of delivery**				
Day	8,675 (42%)	621 (42%)	1,047 (44%)	64 (41%)
Night	12,159 (58%)	847 (58%)	1,329 (56%)	91 (59%)
**Labor analgesia**				
No	17,691 (85%)	1,084 (74%)	1,902 (80%)	93 (60%)
Yes	3,143 (15%)	384 (26%)	474 (20%)	62 (40%)
**GDM**				
No	18,187 (87%)	1,179 (80%)	2,020 (85%)	121 (78%)
Yes	2,647 (13%)	289 (20%)	356 (15%)	34 (22%)
**Umbilical cord around the neck**				
No	14,599 (70%)	1,041 (71%)	1,655 (70%)	102 (66%)
Yes	6,235 (30%)	427 (29%)	721 (30%)	53 (34%)
**PROM**				
No	16,393 (79%)	965 (66%)	1,854 (78%)	86 (55%)
Yes	4,441 (21%)	503 (34%)	522 (22%)	69 (45%)
**Anemia**				
No	17,358 (83%)	1,073 (73%)	2,023 (85%)	116 (75%)
Yes	3,476 (17%)	395 (27%)	353 (15%)	39 (25%)
**Oligohydramnios**				
No	19,769 (95%)	1,392 (95%)	2,239 (94%)	146 (94%)
Yes	1,065 (5.1%)	76 (5.2%)	137 (5.8%)	9 (5.8%)
**Hydramnios**				
No	18,813 (90%)	1,281 (87%)	2,154 (91%)	118 (76%)
Yes	2,021 (9.7%)	187 (13%)	222 (9.3%)	37 (24%)
**Hypertension**				
No	19,441 (93%)	1,255 (85%)	2,208 (93%)	130 (84%)
Yes	1,393 (6.7%)	213 (15%)	168 (7.1%)	25 (16%)
**Adenomyosis**				
No	20,293 (97%)	1,357 (92%)	2,302 (97%)	144 (93%)
Yes	541 (2.6%)	111 (7.6%)	74 (3.1%)	11 (7.1%)
**Placental adhesion**				
No	20,133 (97%)	1,320 (90%)	2,307 (97%)	138 (89%)
Yes	701 (3.4%)	148 (10%)	69 (2.9%)	17 (11%)

*Values are reported as no. (%)*.

*GDM, gestational diabetes mellitus; PROM, premature rupture of membranes*.

### Analysis of Risk Factors for PPH

Univariate and multivariate logistic regression analyses of PPH are presented in Table 2 and revealed that PPH is associated with older age, ethnic minorities, light physical work, first delivery, greater gestational weeks, prolonged delivery time, greater neonatal weight, analgesic delivery, gestational diabetes mellitus, premature rupture of membranes, anemia, hypertension, adenomyosis, and placental adhesion.

**Table 2:** Univariate and multivariate logistic regression analyses of postpartum hemorrhage.

**Characteristic**	**Univariate logistic regression**			**Multivariate logistic regression**		
	**OR**	**95% CI**	***P*-value**	**OR**	**95% CI**	***P*-value**
**Age (years)**						
≤29	Reference			Reference		
30–34	1.34	1.20, 1.50	<0.001^*^	1.35	1.20, 1.53	<0.001^*^
≥35	1.07	0.84, 1.34	0.592	1.53	1.16, 1.99	0.002^*^
**Race**						
Han nationality	Reference			Reference		
Ethnic minorities	1.58	1.35, 1.83	<0.001^*^	1.70	1.44, 2.00	<0.001^*^
**Education**						
Middle school or below	Reference			Reference		
High school	1.21	0.97, 1.54	0.103	0.94	0.73, 1.22	0.635
Undergraduate	1.41	1.14, 1.77	0.002^*^	0.93	0.73, 1.19	0.557
Graduate or above	1.65	1.28, 2.13	<0.001^*^	1.03	0.77, 1.38	0.841
**Occupation**						
Heavy physical	Reference			Reference		
Moderate physical	2.13	1.88, 2.40	<0.001^*^	2.08	1.82, 2.39	<0.001^*^
Light physical	2.28	1.93, 2.70	<0.001^*^	2.27	1.87, 2.74	<0.001^*^
**Pregnancy**						
1	Reference			Reference		
2	0.79	0.69, 0.89	<0.001^*^	0.95	0.82, 1.09	0.467
≥3	0.81	0.68, 0.95	0.012^*^	1.08	0.89, 1.31	0.412
**Parity**						
0	Reference			Reference		
≥1	0.39	0.33, 0.46	<0.001^*^	0.41	0.33, 0.50	<0.001^*^
**Gestational weeks**						
<40	Reference			Reference		
≥40	1.72	1.55, 1.91	<0.001^*^	1.64	1.45, 1.84	<0.001^*^
**Duration of first stage of labor (min)**						
≤303	Reference			Reference		
>303	2.32	2.08, 2.59	<0.001^*^	2.17	1.92, 2.44	<0.001^*^
**Duration of second stage of labor (min)**						
≤68	Reference			Reference		
>68	2.73	2.42, 3.06	<0.001^*^	1.84	1.62, 2.10	<0.001^*^
**Duration of third stage of labor (min)**						
≤9	Reference			Reference		
>9	2.67	2.34, 3.03	<0.001^*^	2.16	1.86, 2.51	<0.001^*^
**Neonatal weight (g)**						
≤3,860	Reference			Reference		
3,861–4,000	1.92	1.55, 2.35	<0.001^*^	1.93	1.54, 2.41	<0.001^*^
>4,000	5.37	4.65, 6.19	<0.001^*^	6.44	5.45, 7.58	<0.001^*^
**Time of delivery**						
Day	Reference			–	–	–
Night	0.97	0.87, 1.08	0.618	–	–	–
**Labor analgesia**						
No	Reference			Reference		
Yes	1.99	1.76, 2.25	<0.001^*^	2.10	1.83, 2.40	<0.001^*^
**GDM**						
No	Reference			Reference		
Yes	1.68	1.47, 1.92	<0.001^*^	1.59	1.37, 1.85	<0.001^*^
**Umbilical cord around the neck**						
No	Reference			–	–	–
Yes	0.96	0.85, 1.08	0.497	–	–	–
**PROM**						
No	Reference			Reference		
Yes	1.92	1.72, 2.15	<0.001^*^	2.27	2.00, 2.58	<0.001^*^
**Anemia**						
No	Reference			Reference		
Yes	1.84	1.63, 2.07	<0.001^*^	2.33	2.03, 2.66	<0.001^*^
**Oligohydramnios**						
No	Reference			–	–	–
Yes	1.01	0.79, 1.28	0.913	–	–	–
**Hydramnios**						
No	Reference			Reference		
Yes	1.36	1.15, 1.59	<0.001^*^	1.80	1.51, 2.15	<0.001^*^
**Hypertension**						
No	Reference			Reference		
Yes	2.37	2.02, 2.76	<0.001^*^	2.96	2.48, 3.52	<0.001^*^
**Adenomyosis**						
No	Reference			Reference		
Yes	3.07	2.47, 3.78	<0.001^*^	3.38	2.66, 4.27	<0.001^*^
**Placental adhesion**						
No	Reference			Reference		
Yes	3.22	2.67, 3.87	<0.001^*^	2.97	2.38, 3.69	<0.001^*^

*GDM, gestational diabetes mellitus; PROM, premature rupture of membranes; OR, Odds Ratio; CI, Confidence Interval; Ref, Reference*.

* *P < 0.05*.

### Nomogram Construction

**Figure 1:**
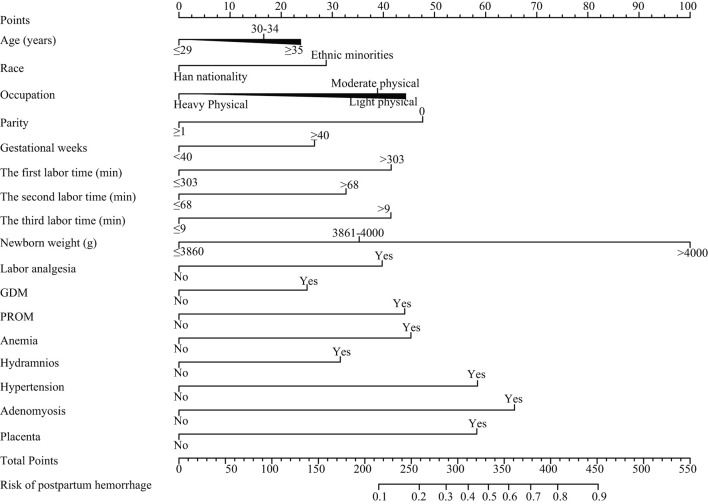
Nomograms for postpartum hemorrhage. GDM, gestational diabetes mellitus; PROM, premature rupture of membranes.

**Figure 2:**
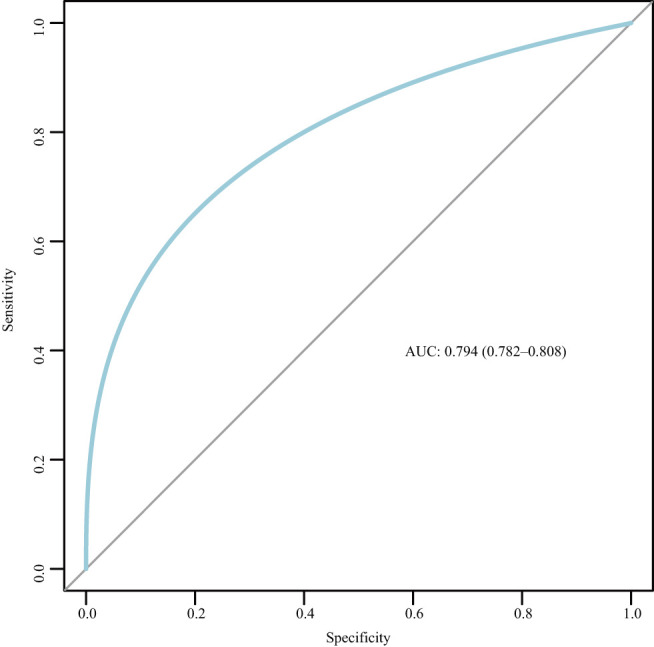
Receiver operating characteristic (ROC) curve for the nomogram. AUC, area under the curve; ROC, receiver operating characteristic.

**Figure 3:**
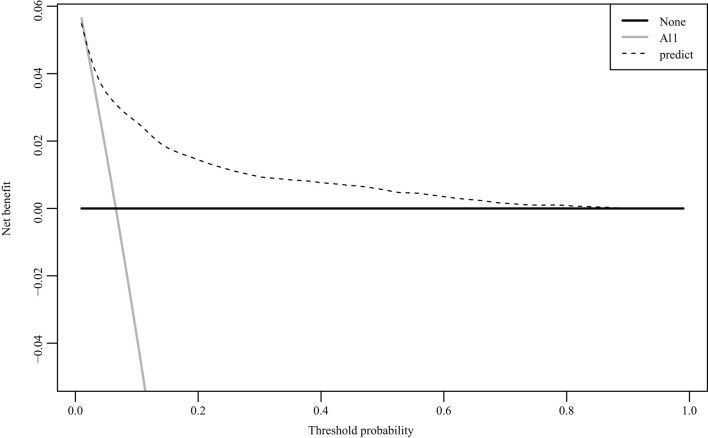
Decision curve analysis curve for the nomogram.

**Figure 4:**
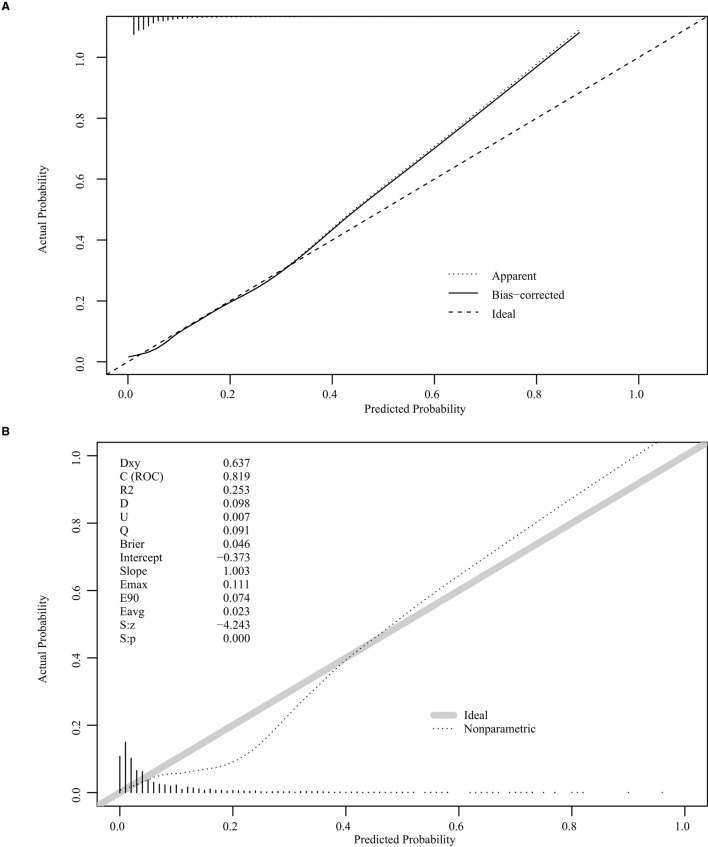
Internal and external validation plots of the nomogram calibration curves. **(A)** Internal validation; **(B)** External validation.

The nomogram was based on the important variables of the multivariate logistic regression analysis (Figure 1). The ROC curve of the nomograms used to evaluate PPH is shown in Figure 2. The nomogram's AUC was higher than 75% using the variables selected in multiple logistic regression, indicating that the nomogram can effectively predict PPH. In addition, DCA (Figure 3) suggests that the nomogram has potential clinical benefit. The nomogram was internally verified by bootstrapping 1,000 resampling, and the calibration curves were close to the 45° line, suggesting that the nomogram's prediction probability was consistent with the actual probability (Figure 4A). The nomogram was verified using an external validation cohort, and the calibration curve was close to 45°, indicating consistency between the predicted probability and the actual probability in the external validation cohort (Figure 4B).

## Discussion

PPH is usually defined as blood loss of at least 500 mL following vaginal delivery or 1,000 mL following a cesarean section within 24 h postpartum (12, 13). Due to the lack of a global ‘gold standard' for accurately measuring PPH and the unreliability of visual estimation of PPH in clinical practice, reports on the incidence of PPH vary among countries and regions, ranging from 1 to 11% (14). A large study from the United States revealed a 2.6% increase in PPH cases and a 5.3% increase in the incidence of PPH between 1994 and 2006 (15). Feduniw et al. reported the incidence of PPH in different regions around the world, ranging from 0.3 to 3.8% in Africa, 0.7 to 2.7% in Asia, and 1.7 to 5.5% in Europe (14). The most commonly used measurement method is visual estimation, which is associated with a high possibility error, so the actual incidence of PPH should be higher than that reported locally. Underestimation occurs in 30–50% if it is only visual (12).

With the changes in social concepts and lifestyles, the age of female childbearing has changed, with increased incidence of advanced maternal age (16). In a cohort study of 103,726 pregnant women published by Kramer in 2011, age ≥35 years was weakly associated with PPH [odds ratio (OR): 1.5 (1.5, 1.6)] (17). In 2014, Oberg et al. published a large sample cohort involving 900 000 pregnant women, which also obtained similar results with a combined OR value of 2 (1.9, 2.2). The most influential continuous variable was newborn weight, according to the nomogram. This result is consistent with Ononge's report that macrosomia is a risk factor for PPH (18). The larger fetus may lead to uterine muscle fiber damage, causing uterine contraction weakness, resulting in bleeding; On the other hand, a larger fetus can lead to delay of the fetal head entering the basin or blockage of internal rotation, resulting in prolonged labor and increased bleeding (19).

Prolonged or stagnant labor can easily lead to damage to the soft birth canal, weak uterine contractions, and increased PPH rates. Le Ray et al. (20) reported that the crude OR (95% CI) was 1.9 (1.0–3.7) when the duration of active first stage was 4–6 h and the crude OR (95% CI) was 1.3 (0.7–2.2) when the duration of passive second stage was 1–2 h. Magann et al. (21) reported that when the third stage of labor exceeds 15 min, it is a key risk factor for PPH. Dixon et al. (22) and Sosa et al. (23) both reported that actively managing the third stage of labor and reducing the duration of the third stage can reduce the probability of PPH.

Studies have demonstrated that maternal anemia before delivery is an independent risk factor for PPH (24). Oyelese et al. (25) reported that severe anemia may lead to myoedema of the uterus and decreased resistance to infectious diseases, resulting in weak uterine contractions and uterine muscle dysfunction, ultimately leading to PPH. In addition, according to the PPH guidelines of the Society of Obstetricians and Gynecologists of Canada, the risk of PPH increases when the hemoglobin level is below 80 g/L, and the risk of PPH decreases by 0.86 for every 19 g/L increase in Hb (95% CI: 0.78–0.90) (26).

Hyperhydramnios is an independent risk factor for PPH. Its pathogenic factors are similar to those of macrosomia, which causes excessive stretching of the maternal uterine muscle fibers, which then affects the uterine contractility, causing PPH. The same principle applies to uterine fibroids and myoadenosis. Noor et al. (27) and Olive et al. (28) both reported that pregnancy complicated with uterine fibroids increased the incidence of PPH. Large uterine fibroids can also cause uterine weakness and uncoordinated contractions after labor, resulting in abnormal fetal position and prolonged labor (29). In addition, the low growth position of uterine fibroids can cause low-lying or preposition of the placenta, which can lead to PPH (30). Patients with gestational diabetes are prone to hyperenlargement of the uterus due to macrocephaly or hydramnios, resulting in reduced uterine contractility, which often leads to an increased risk of PPH (18). Similarly, uterine contraction and coagulation function can be affected by inflammatory disorders complicating pregnancy and sedation, spasmolysis, and antihypertensive agents may relax the smooth muscle tissue of the uterus, thereby affecting uterine contraction (14).

Although some researches about labor analgesia have shown that epidural labor analgesia does not increase the risk of vaginal labor PPH (31, 32), our study still demonstrated a relationship between labor pain relief and PPH outcome. The predicted risk of PPH is higher in patients who received labor analgesia than in those who did not, which may be associated with prolonged labor or lack of contractions. The results also show that physical activity level was correlated with PPH. It may be due to physical labor during pregnancy, strengthen the strength of pelvic floor muscles, so that the rotation and decline of the fetal head in the process of delivery is relatively smooth, and the labor process is relatively shortened.

In general, PPH is affected by many factors, including abnormal coagulation, improper use of uterine contraction agents, or simple weak uterine contractions. This study only analyzed clinical pregnancy-related diseases and objective factors before and during delivery and did not include laboratory examination results as predictive indicators, which is a limitation. In addition, although the measurement method of PPH was strictly defined, it was difficult to maintain the measurement standard for each delivery under the management of many different doctors because it was a retrospective study, which is one of the limitations of this study. Clinical usefulness is an important indicator to determine whether a predictive model can truly be applied clinically to benefit patients. The DCA curve can be used to determine whether the benefits of the model outweigh the disadvantages in clinical use. In addition to the traditional ROC curve, we also used the DCA curve to evaluate the nomogram's performance (33). In our prediction model, when the threshold probability was between 0 and 90%, the net benefits of the nomogram were better than those of the scenarios in which all patients had PPH or none had PPH.

## Conclusion

This nomogram can be used to predict PPH after vaginal delivery. Therefore, it can be used to screen high-risk patients in clinical practice and enable clinicians to make appropriate interventions in advance to mitigate against PPH. This could be of great benefit because of the high global incidence of PPH and the potential to impact morbidity and mortality of patients.

## Data Availability Statement

The original contributions presented in the study are included in the article/Supplementary Material, further inquiries can be directed to the corresponding author/s.

## Ethics Statement

The studies involving human participants were reviewed and approved by the Ethics Committee of the Shengjing Hospital of China Medical University. The patients/participants provided their written informed consent to participate in this study.

## Author Contributions

ZS and DZ designed the study and drafted the manuscript. XW, YW, and YZ designed the statistical analysis plan. DZ reviewed the manuscript. All authors take responsibility for the appropriateness of the content. All authors contributed to the article and approved the submitted version.

## Funding

This research was supported by internal funding from Shengjing Hospital, China Medical University (SJ-M0133) and 345 Talent Project of Shengjing Hospital of China Medical University (No. M0946).

## Conflict of Interest

The authors declare that the research was conducted in the absence of any commercial or financial relationships that could be construed as a potential conflict of interest.

## Publisher's Note

All claims expressed in this article are solely those of the authors and do not necessarily represent those of their affiliated organizations, or those of the publisher, the editors and the reviewers. Any product that may be evaluated in this article, or claim that may be made by its manufacturer, is not guaranteed or endorsed by the publisher.

## Supplementary Material

Supplementary Material S1 Analysis of X-tile software for age.

Supplementary Material S2 Analysis of X-tile software for the duration of the first stage of labor.

Supplementary Material S3 Analysis of X-tile software for duration of the second stage of labor.

Supplementary Material S4 Analysis of X-tile software for the third stage of labor.

Supplementary Material S5 Analysis of X-tile software for neonatal weight.
