# Supplementary material for: Development and Validation of Prognostic Nomogram for Postpartum Hemorrhage After Vaginal Delivery: A Retrospective Cohort Study in China
Source: Front Med (Lausanne). 2022 Mar 7;9:804769. doi: 10.3389/fmed.2022.804769 (PMC8936128; doi:10.3389/fmed.2022.804769)
Supplement: Supplementary Material S1 — Analysis of X-tile software for age. [file Data_Sheet_1.PDF]

Survival Analysis: Age

2021年8月24日 16:44:28
lenovo

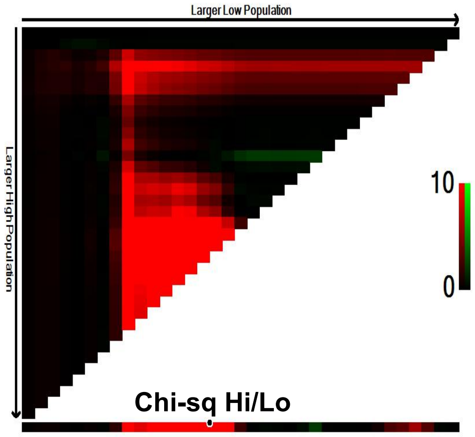

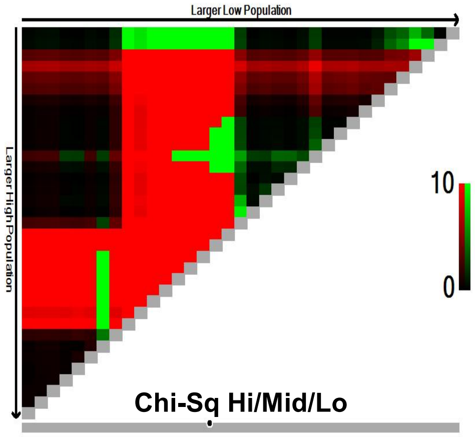

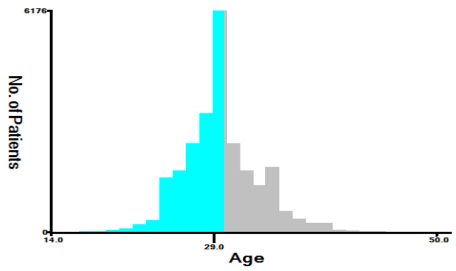

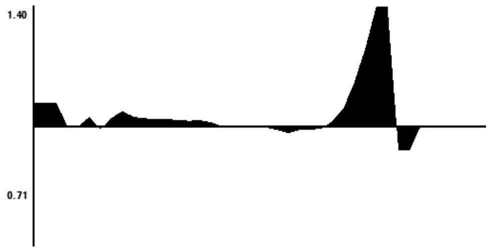

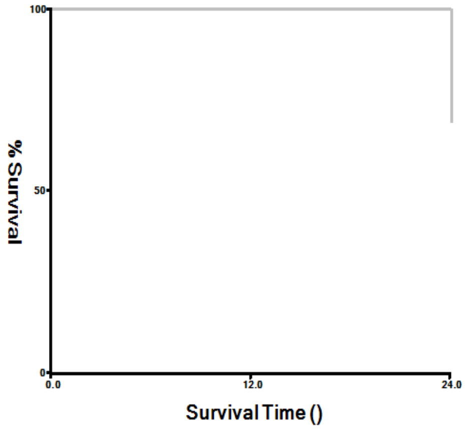

Subpopulation Cutpoints:

Pt No	% Total	Events	Rate	Rank	Range
13045	52.53	747	5.73	0 to 15	14.00 thru 29.00
11788	47.47	876	7.43	16 to 35	30.00 thru 50.00
24833	100.00	1623	6.54	0 to 35	14.00 thru 50.00

Statistics:

Variable	Value	
Miller-Seigmund P	<0.0001	Max: <0.0001
Chi-sq Hi/Lo	51.2588	Max: 51.2588
Relative Risk 1 vs 2	1.00 / 1.30	

Copyright: Camp/Rimm, Yale University
www.tissuearray.org
