# Supplementary material for: Development and Validation of Prognostic Nomogram for Postpartum Hemorrhage After Vaginal Delivery: A Retrospective Cohort Study in China
Source: Front Med (Lausanne). 2022 Mar 7;9:804769. doi: 10.3389/fmed.2022.804769 (PMC8936128; doi:10.3389/fmed.2022.804769)
Supplement: Supplementary Material S2 — Analysis of X-tile software for the duration of the first stage of labor. [file Data_Sheet_2.PDF]

Survival Analysis: T1

2021年8月24日 16:47:55
lenovo

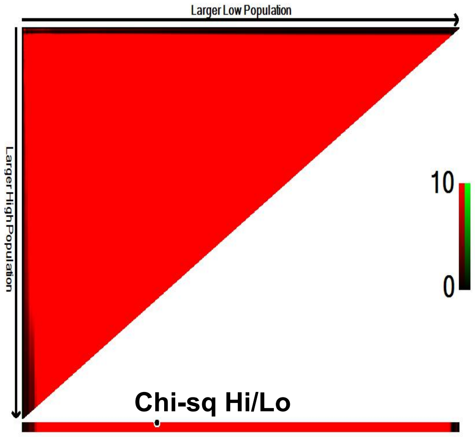

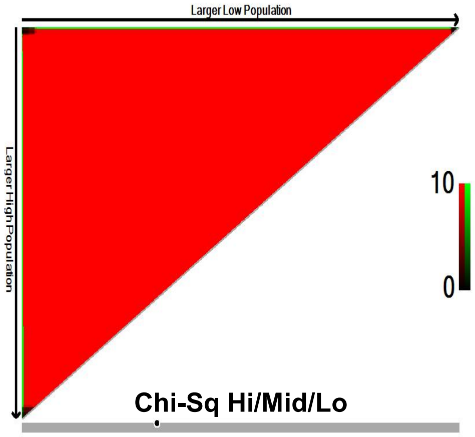

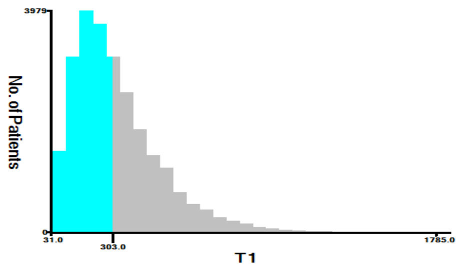

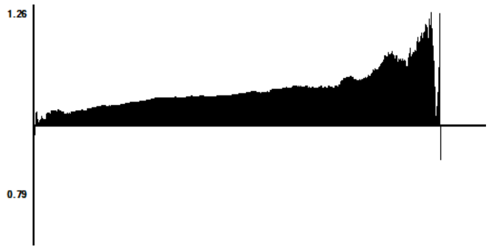

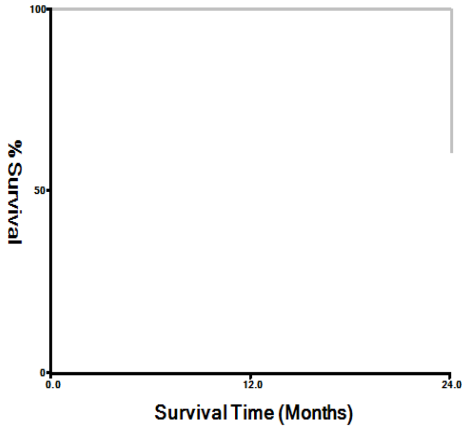

Subpopulation Cutpoints:

Pt No	% Total	Events	Rate	Rank	Range
13968	56.25	597	4.27	0 to 264	31.00 thru 303.00
10865	43.75	1026	9.44	265 to 856	304.00 thru 1785.00
24833	100.00	1623	6.54	0 to 856	31.00 thru 1785.00

Statistics:

Variable	Value	
Miller-Seigmund P	<0.0001	Max: <0.0001
Chi-sq Hi/Lo	280.4677	Max: 280.4677
Relative Risk 1 vs 2	1.00 / 2.21	

Copyright: Camp/Rimm, Yale University
www.tissuearray.org
