# Supplementary material for: Development and Validation of Prognostic Nomogram for Postpartum Hemorrhage After Vaginal Delivery: A Retrospective Cohort Study in China
Source: Front Med (Lausanne). 2022 Mar 7;9:804769. doi: 10.3389/fmed.2022.804769 (PMC8936128; doi:10.3389/fmed.2022.804769)
Supplement: Supplementary Material S3 — Analysis of X-tile software for duration of the second stage of labor. [file Data_Sheet_3.PDF]

Survival Analysis: T2

2021年8月24日 16:48:52
lenovo

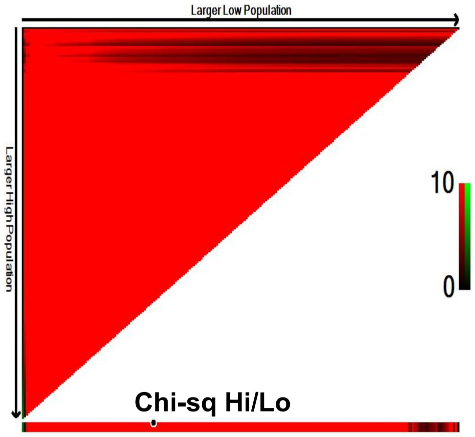

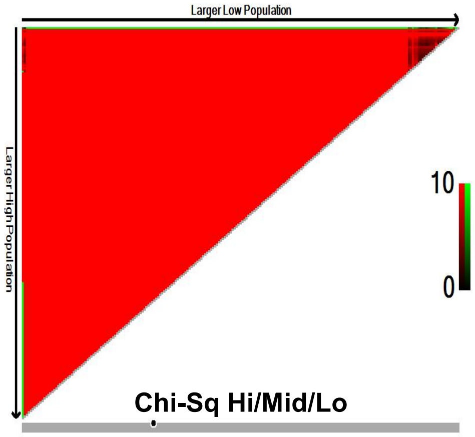

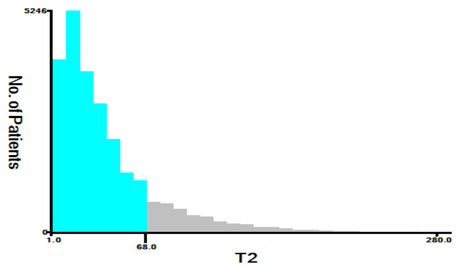

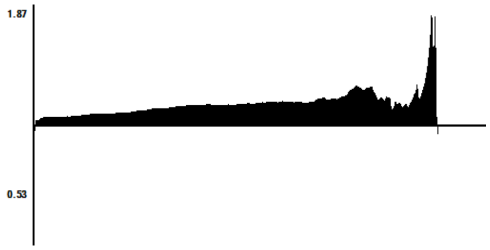

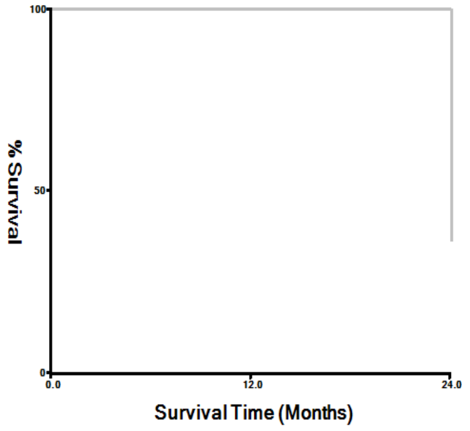

Subpopulation Cutpoints:

Pt No	% Total	Events	Rate	Rank	Range
21013	84.62	1108	5.27	0 to 67	1.00 thru 68.00
3820	15.38	515	13.48	68 to 223	69.00 thru 280.00
24833	100.00	1623	6.54	0 to 223	1.00 thru 280.00

Statistics:

Variable	Value	
Miller-Seigmund P	<0.0001	Max: <0.0001
Chi-sq Hi/Lo	350.0328	Max: 350.0328
Relative Risk 1 vs 2	1.00 / 2.56	

Copyright: Camp/Rimm, Yale University
www.tissuearray.org
