# Supplementary material for: Development and Validation of Prognostic Nomogram for Postpartum Hemorrhage After Vaginal Delivery: A Retrospective Cohort Study in China
Source: Front Med (Lausanne). 2022 Mar 7;9:804769. doi: 10.3389/fmed.2022.804769 (PMC8936128; doi:10.3389/fmed.2022.804769)
Supplement: Supplementary Material S4 — Analysis of X-tile software for the third stage of labor. [file Data_Sheet_4.PDF]

Survival Analysis: T3

2021年8月24日 16:49:14
lenovo

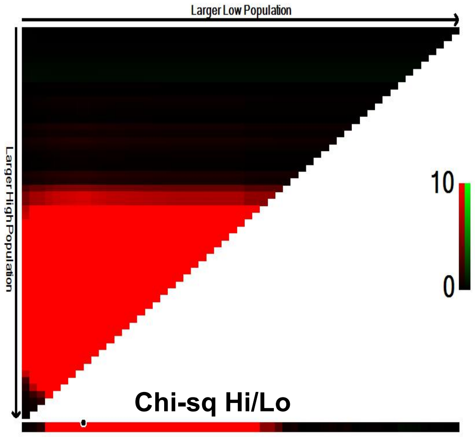

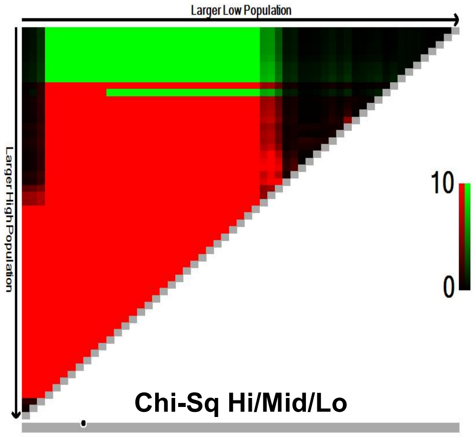

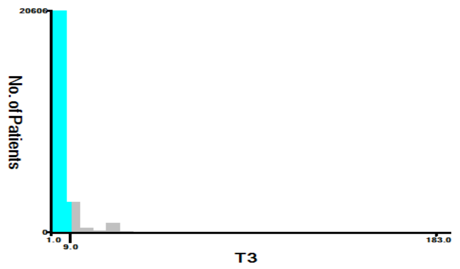

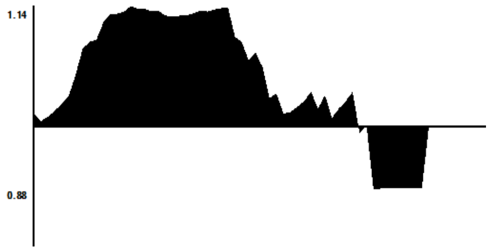

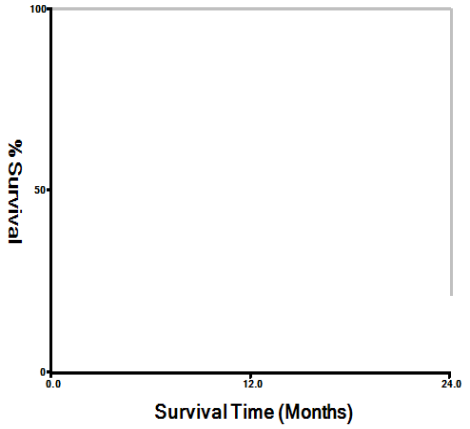

Subpopulation Cutpoints:

Pt No	% Total	Events	Rate	Rank	Range
22054	88.81	1236	5.60	0 to 8	1.00 thru 9.00
2779	11.19	387	13.93	9 to 57	10.00 thru 183.00
24833	100.00	1623	6.54	0 to 57	1.00 thru 183.00

Statistics:

Variable	Value	
Miller-Seigmund P	<0.0001	Max: <0.0001
Chi-sq Hi/Lo	252.8361	Max: 252.8361
Relative Risk 1 vs 2	1.00 / 2.48	

Copyright: Camp/Rimm, Yale University
www.tissuearray.org
