# Supplementary material for: Development and Validation of Prognostic Nomogram for Postpartum Hemorrhage After Vaginal Delivery: A Retrospective Cohort Study in China
Source: Front Med (Lausanne). 2022 Mar 7;9:804769. doi: 10.3389/fmed.2022.804769 (PMC8936128; doi:10.3389/fmed.2022.804769)
Supplement: Supplementary Material S5 — Analysis of X-tile software for neonatal weight. [file Data_Sheet_5.PDF]

Survival Analysis: Weight

2021年8月24日 16:50:48
lenovo

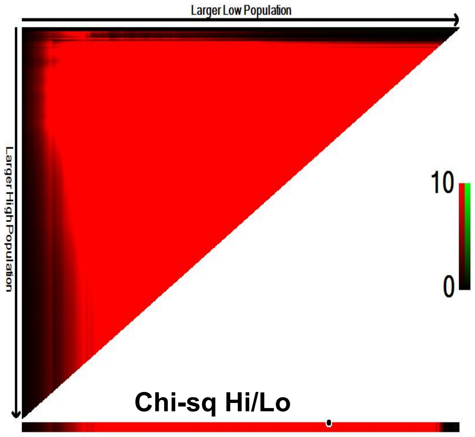

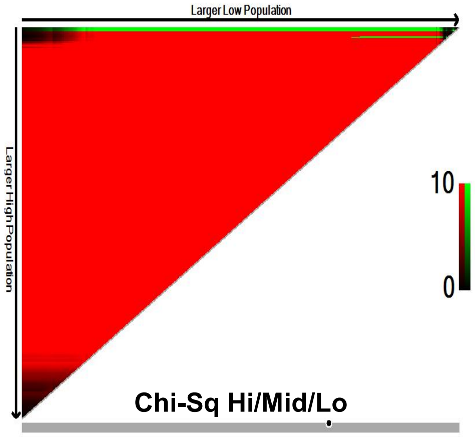

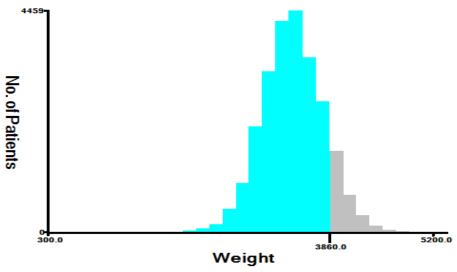

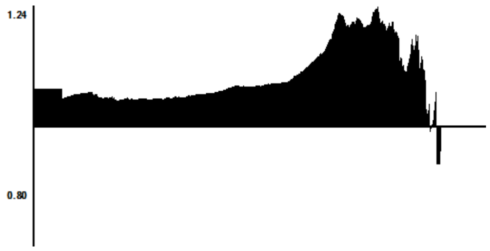

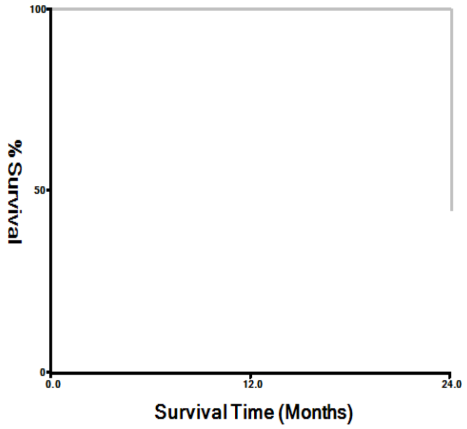

Subpopulation Cutpoints:

Pt No	% Total	Events	Rate	Rank	Range
22179	89.31	1171	5.28	0 to 391	300.00 thru 3860.00
2654	10.69	452	17.03	392 to 557	3865.00 thru 5200.00
24833	100.00	1623	6.54	0 to 557	300.00 thru 5200.00

Statistics:

Variable	Value	
Miller-Seigmund P	<0.0001	Max: <0.0001
Chi-sq Hi/Lo	521.3640	Max: 521.3640
Relative Risk 1 vs 2	1.00 / 3.23	

Copyright: Camp/Rimm, Yale University
www.tissuearray.org
